# Associations of multiple carotenoid co-exposure with all-cause and cause-specific mortality in US adults: a prospective cohort study

**DOI:** 10.3389/fnut.2024.1415537

**Published:** 2024-08-07

**Authors:** Qinglin He, Chunling Yuan, Zhihui Liu, Xiaoxia Wei

**Affiliations:** ^1^Day Oncology Unit, Guangxi Medical University Cancer Hospital, Nanning, China; ^2^Department of Medical Quality Control, Guangxi Medical University Cancer Hospital, Nanning, China; ^3^Department of Clinical Research, Guangxi Medical University Cancer Hospital, Nanning, China

**Keywords:** NHANES, carotenoid, co-exposure, K-means, mortality

## Abstract

**Background:**

Epidemiological evidence regarding circulating carotenoids and mortality risk remains conflicting, and most studies focus on the impact of individual carotenoids. This study aimed to elucidate the effects of co-exposure to multiple serum carotenoids on mortality risk.

**Methods:**

We enrolled 22,472 participants aged ≥20 from the National Health and Nutrition Examination Survey (NHANES) III (1988–1994) and NHANES 2003–2006. Baseline serum levels of five major carotenoids (α-carotene, β-carotene, lycopene, β-cryptoxanthin, and lutein/zeaxanthin) were measured, and individuals were followed up until December 31, 2019. Carotenoid co-exposure patterns were identified using the K-means method. Cox proportional hazard models were used to investigate the associations between carotenoid exposure and mortality risk.

**Results:**

During a median follow-up of 16.7 years, 7,901 deaths occurred. K-means clustered participants into low-level, low-lycopene, high-lycopene, and high-level exposure groups. In the fully adjusted model, low-lycopene, high-lycopene, and high-level exposure groups had significantly lower all-cause mortality risks compared to the low-level exposure group, with hazard ratios (HRs) and 95% confidence intervals (CIs) of 0.79 (0.72, 0.87), 0.75 (0.67, 0.84), and 0.67 (0.61, 0.74), respectively. For cardiovascular disease mortality, the high-lycopene exposure group had a 27% reduced risk (HR: 0.73, 95% CI: 0.61–0.86), and the high-level exposure group had a 21% reduced risk (HR: 0.79, 95% CI: 0.67–0.93). For cancer mortality, the high-lycopene and high-level exposure groups had 30% and 35% lower risks, with HRs (95% CIs) of 0.70 (0.57, 0.86) and 0.65 (0.54, 0.79), respectively.

**Conclusion:**

This study revealed that co-exposure to multiple serum carotenoids was associated with reduced mortality risk, highlighting the potential health benefits of increased carotenoid intake. Further investigation is warranted to elucidate the underlying mechanisms of interactions among different carotenoids.

## 1 Introduction

Carotenoids are essential micronutrients present in food, synthesized by photosynthetic organisms and certain microorganisms (1). Over 700 carotenoids have been identified, but only 40 are commonly consumed in the human diet. The most common include α-carotene, β-carotene, lycopene, β-cryptoxanthin, lutein, and zeaxanthin (2). Carotenoids are vital for maintaining health by neutralizing free radicals and reducing oxidative stress, which can contribute to chronic diseases such as cardiovascular disease (CVD) and cancer (3). For instance, lycopene has been extensively studied for its protective effects against CVD due to its strong singlet oxygen-quenching ability (4). β-carotene can reduce cancer risk by mitigating oxidative stress and enhancing the body’s antioxidant defense system (5). Beyond their well-documented antioxidative functions, carotenoids have demonstrated anti-inflammatory, anticancer, and immune system regulatory properties in laboratory research (6–8). These properties may contribute to their beneficial effects on health outcomes (9).

Current studies on the effects of carotenoids on human health have yielded inconsistent results. Several epidemiological studies have demonstrated that increased circulating levels of carotenoids are associated with a decreased incidence of CVD (10, 11) and lower mortality risk (12–15). However, some observational studies and interventions involving carotenoid supplementation, particularly β-carotene, have shown either neutral or adverse effects on all-cause and CVD mortality across various populations (16, 17). This discrepancy may be due to differences in study design, population characteristics, carotenoid bioavailability, and confounding factors (18). Additionally, most published studies have focused on individual carotenoid exposures (19–22) without considering how the efficacy of one carotenoid might be influenced by the presence of others (9). For example, the bioavailability and function of lutein can be influenced by the presence of other carotenoids, such as lycopene and β-carotene (23). Consequently, it has been challenging to assess the combined effects of multiple carotenoid co-exposure on mortality risk in these studies.

Humans are commonly exposed to multiple carotenoids simultaneously in daily life, potentially resulting in interactions among them (9). For instance, significant interactions between lycopene and lutein/zeaxanthin have been observed in modulating health outcomes (14). Considering the complex interplay between serum carotenoids, a multivariate approach is necessary to understand their joint effects on health. Therefore, in this study, we employed an unsupervised clustering (k-means) method to develop co-exposure patterns of carotenoids and comprehensively explore the associations of these patterns with mortality risk in a nationally representative sample of U.S. adults. This approach allows us to address the gap in the literature regarding the combined effects of multiple carotenoids, providing scientific and practical guidance for reducing mortality risk through optimized carotenoid intake.

## 2 Materials and methods

### 2.1 Study design and participants

The National Health and Nutrition Examination Survey (NHANES) combines interviews and physical examinations to evaluate adults’ and children’s health and nutritional status across the United States. Conducted by the National Center for Health Statistics (NCHS), NHANES requires all participants to provide written informed consent and has obtained approval from the NCHS Ethics Review Board (24).

**Figure 1:**
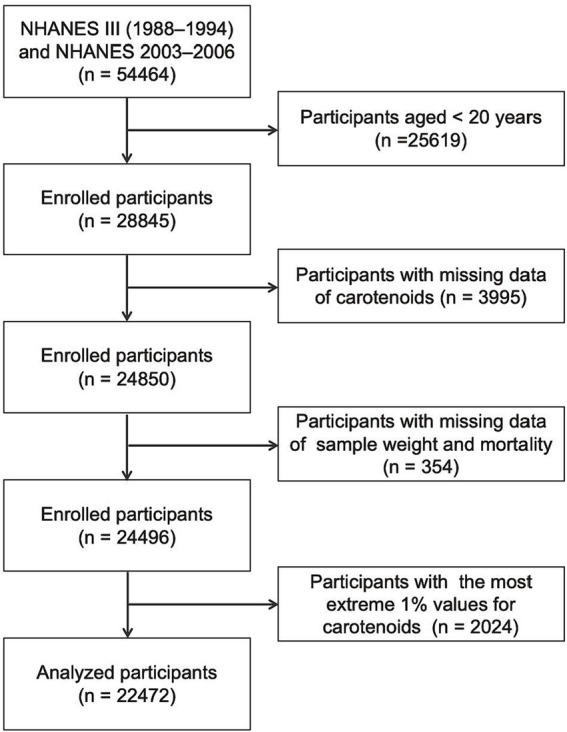
Study design overview.

This study included participants aged 20 years and older in NHANES III (1988–1994) and continuous NHANES (2003–2006) surveys, as only participants from these periods provided data on the five primary serum carotenoids. Participants with missing data on serum carotenoid levels, sample weight, or mortality were excluded. Additionally, those with the most extreme 1% values for the five serum carotenoids were excluded. Ultimately, 22,472 participants were retained for the final analysis (Figure 1).

### 2.2 Measurement of carotenoids

Serum concentrations of α-carotene, β-carotene, lycopene, β-cryptoxanthin, and lutein/zeaxanthin were determined via high-performance liquid chromatography (HPLC) in NHANES III and NHANES 2005–2006. In NHANES 2003–2004, a similar HPLC method was employed for measuring these carotenoids. Consequently, data from NHANES 2003–2004 were converted to equivalent carotenoid measurements from the HPLC method using a regression method. Detailed laboratory procedures and quality control methods for serum carotenoid measurements are described at: https://wwwn.cdc.gov/nchs/nhanes/continuousnhanes/labmethods.aspx?Cycle=2003-2004.

### 2.3 Ascertainment of outcomes

Mortality status was determined by linking to National Death Index records up to December 31, 2019.^1^ In our analysis, we used the International Classification of Diseases, Tenth Revision (ICD-10) codes to define the primary outcomes: death from all causes, CVD (I00–I09, I11, I13, I20–I51, and I60–I69), and cancer (C00–C97). The follow-up period was calculated from the NHANES interview date until death, loss to follow-up, or censoring (December 31, 2019), whichever came first.

### 2.4 Assessment of covariates

Demographic and lifestyle data were collected from baseline household questionnaires and used as covariates. These data included age, sex, race, family income-poverty ratio (FIPR), education level, body mass index (BMI), marital status, smoking status, drinking status, and histories of hypertension, high cholesterol, diabetes, CVD, and cancer. Participants were categorized as never smokers, former smokers, or current smokers based on their responses to questions about smoking at least 100 cigarettes during their lifetime and current smoking status. Drinking status was categorized as non-drinker or drinker based on alcohol consumption of at least 12 times a year. Hypertension was defined by an average systolic pressure of ≥140 mmHg, an average diastolic pressure of ≥90 mmHg, ongoing antihypertensive treatment, or a self-reported physician diagnosis. High cholesterol was indicated by total cholesterol levels of ≥240 mg/dL, self-reported use of prescribed cholesterol-lowering medication, or a self-reported physician diagnosis of high cholesterol. Participants were considered to have CVD if they had a history of coronary heart disease, angina/chest pain, heart attack, congestive heart failure, or stroke. Diabetes was identified through the following criteria: self-reported doctor diagnosis, use of oral hypoglycemic medication or insulin, fasting blood glucose ≥126 mg/dL (7.0 mmol/L), 2-h postprandial plasma glucose ≥200 mg/dL (11.1 mmol/L) from an oral glucose tolerance test, or glycated hemoglobin A1c (HbA1c) levels ≥6.5% (25). Additionally, the questionnaires on cancer history were used to ascertain the presence of cancer.

### 2.5 Statistical analysis

Following the analysis guidelines of the NHANES survey, sample weights were applied. Quantitative data were assessed for normality. Normally distributed data were presented as mean ± standard deviation (SD) and analyzed using ANOVA for intergroup comparisons, while non-normally distributed data were expressed as median (interquartile range, IQR) and analyzed using the Wilcoxon rank-sum test. Qualitative data were presented as frequencies and percentages and analyzed using the Rao-Scott chi-square test.

To improve data normality, carotenoid concentrations were natural log-transformed. Missing data on covariates were encoded as missing indicators for categorical variables and replaced with median values for continuous variables. Pearson correlation coefficients were calculated to evaluate correlations among serum concentrations of five carotenoids. Subsequently, the K-means method was employed to classify participants into distinct clusters based on the standardized serum concentrations of the five carotenoids. The K-means algorithm is a non-model-based method of categorizing mixed data (26). It constructs clusters such that the squared Euclidean distance between the row vector for any object and the centroid vector of its respective cluster is minimized compared to the distances to the centroids of the remaining clusters (27). The optimal number of clusters was determined using the elbow method (27).

The Cox proportional hazards model was utilized to assess the association of serum carotenoids with the risks of all-cause, CVD, and cancer mortality. Hazard ratios (HRs) and their corresponding 95% confidence intervals (CIs) were computed across three models. The proportional hazards assumption was assessed using Schoenfeld residuals, and no violations were observed. Model 1 was adjusted for age (continuous), sex (male or female), race (non-Hispanic White, non-Hispanic Black, Mexican American, or other), and FIPR (0–0.99, ≥ 1, or unknown). Model 2 included further adjustments for education level (below high school, high school, above high school, or unknown), BMI (< 25, 25–30, ≥30, or unknown), marital status (married, other, or unknown), smoking status (never, former, current, or unknown), and drinking status (non-drinker, drinker, or unknown). Model 3 incorporated additional adjustments for histories of hypertension (no, yes, or unknown), high cholesterol (no, yes, or unknown), diabetes (no or yes), CVD (no, yes, or unknown), and cancer (no, yes, or unknown).

To elucidate the relationship between individual carotenoid exposure and mortality, carotenoid concentrations were initially treated as continuous variables, and HRs for one SD unit were estimated. Each carotenoid concentration was then categorized into quartiles. The linear trend test across ascending carotenoid groups was computed using integer values (1, 2, 3, and 4). Three-knot restricted cubic splines (RCS) were fitted to estimate exposure-response curves for serum carotenoid concentrations and mortality risk. In addition, we examined the association between carotenoid co-exposure and population mortality using a categorical model derived from clusters generated by the K-means algorithm.

Sensitivity analyses excluded participants with missing covariate data and those who died within the first 2 years of follow-up. All analyses were performed using R software version 4.3.2 (R foundation, Vienna, Austria). Statistical tests were two-sided, and a significance level of *P* < 0.05 was considered statistically significant.

## 3 Results

### 3.1 Baseline characteristics of participants

After excluding participants with missing data on exposure variables, study outcomes, and major covariates, the study included a total of 22,472 participants. During a median follow-up of 16.7 years (IQR: 13.7–27.3 years), 7,901 deaths were recorded, of which 2,871 were attributed to CVD and 1,717 to cancer.

**Figure 2:**
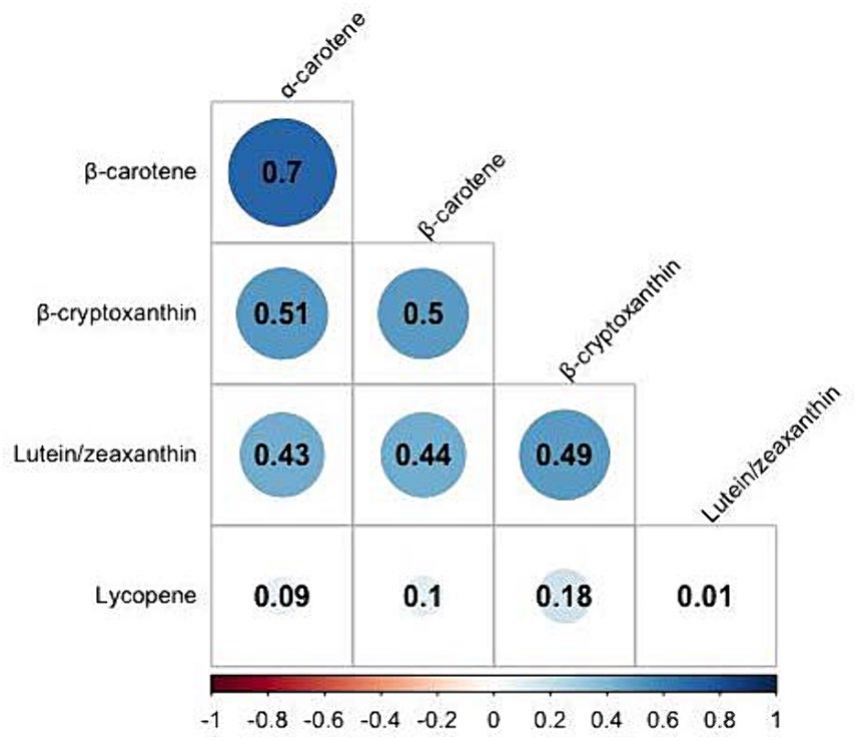
Pearson correlation analysis on serum concentrations of five carotenoids in NHANES III and NHANES 2003–2006.

**Figure 3:**
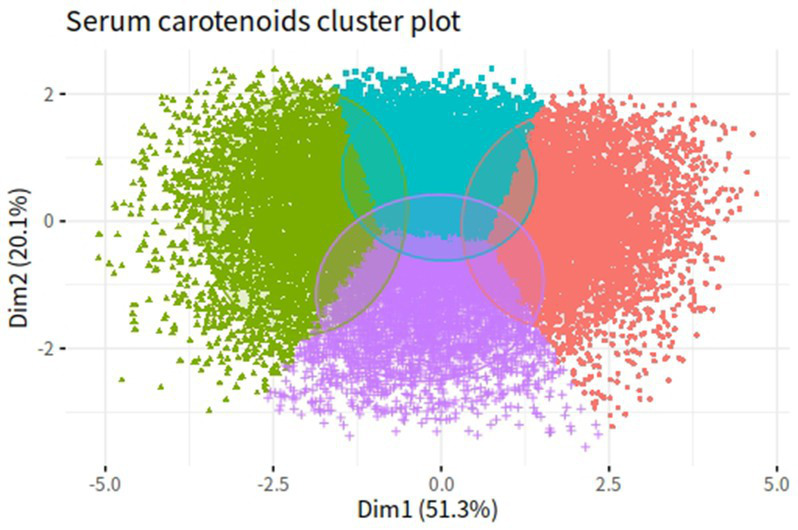
K-means clustering results of five serum carotenoids in NHANES III and NHANES 2003–2006. Green dots represent cluster 1 (low-level exposure group); purple dots represent cluster 2 (low-lycopene exposure group); blue dots represent cluster 3 (high-lycopene exposure group); red dots represent cluster 4 (high-level exposure group).

**Figure 4:**
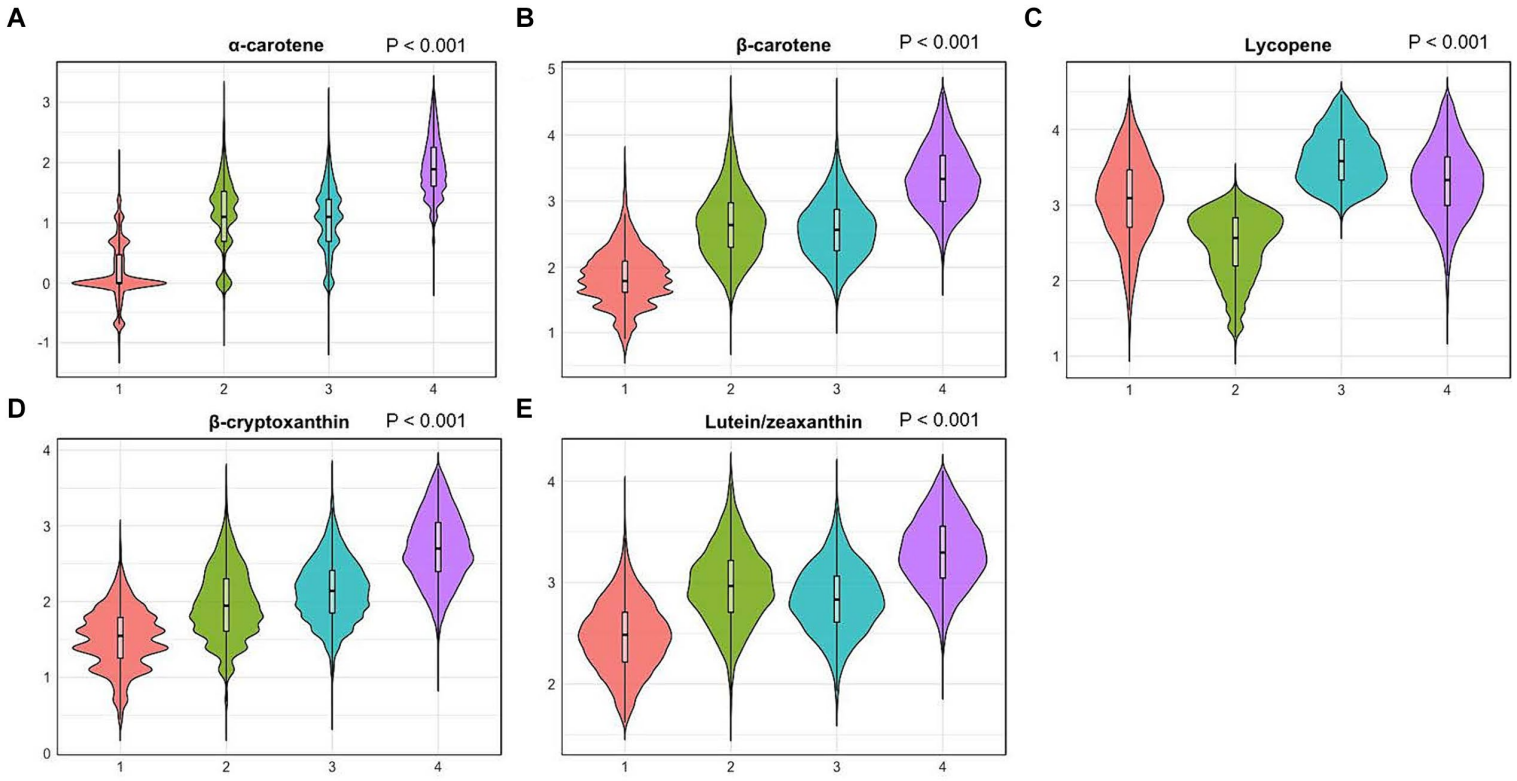
Violin plots of serum carotenoid concentrations in different clusters. **(A)**, violin plots of serum α-carotene; **(B)**, violin plots of serum β-carotene; **(C)**, violin plots of serum lycopene; **(D)**, violin plots of serum β-cryptoxanthin; **(E)**, violin plots of serum lutein/zeaxanthin. Differential analyses were conducted using the sampling-weighted analysis of the Wilcoxon rank-sum test. The X-axis indicates four clusters: 1. Cluster 1 (low-level exposure group); 2. Cluster 2 (low-lycopene exposure group); 3. Cluster 3 (high-lycopene exposure group); 4. Cluster 4 (high-level exposure group). The Y-axis represents standardized serum carotenoid concentrations.

Correlation coefficients between each pair of studied carotenoid measurements ranged from 0.01 to 0.7 (Figure 2). The highest correlation was found between α-carotene and β-carotene, with a correlation coefficient of 0.7. K-means clustering analysis categorized the 22,472 participants into four clusters based on the levels of five serum carotenoids (Figure 3). Supplementary Table S1 presents the distributions of standardized carotenoid concentrations, and Supplementary Table S2 shows the centers of the four clusters. We categorized cluster 1 as the ‘low-level exposure group’ because all five serum carotenoid concentrations were at or below their 25th percentiles. Similarly, we defined cluster 4 as the ‘high-level exposure group’ since all five serum carotenoid concentrations were at or above their 75th percentiles. Cluster 2 had low lycopene levels but moderate levels of the other four carotenoids, which was categorized as the ‘low-lycopene exposure group’. Cluster 3 was labeled as the ‘high-lycopene exposure group’ because participants had high lycopene levels and moderate levels of the other carotenoids. Figure 4 displays the violin plot illustrating the features of the four clusters.

Table 1 presents the baseline characteristics of the study participants within the four clusters. Significant differences were observed in almost all variables among the clusters, except for a history of cancer. Individuals in cluster 1 tended to be younger (median age: 40 years), obese (39.95%), and current smokers (41.97%) compared to those in the other clusters. In contrast, a higher proportion of participants in cluster 2 were older (median age: 52 years), former smokers (31.38%), and had histories of hypertension (39%), diabetes (23.53%), CVD (31.78%), and cancer (7.45%). Non-Hispanic White people comprised more enormous proportions than participants from other ethnic groups across all four clusters, particularly in clusters 1 (76.79%) and 3 (75.00%).

**Table 1:** Baseline characteristics of participants in this study.

Characteristics	Overall (*n* = 22,472)	Cluster 1^a^ (*n* = 5,119)	Cluster 2^b^ (*n* = 4,653)	Cluster 3^c^ (*n* = 6,471)	Cluster 4^d^ (*n* = 6,229)	*p***-**value^e^
Age (years)	43.00 (32.00, 58.00)	40.00 (29.00, 52.00)	52.00 (37.00, 69.00)	42.00 (31.00, 54.00)	47.33 (36.00, 63.00)	<0.001
Sex						<0.001
Male	10,593.00 (48.02%)	2,610.00 (52.26%)	2,151.00 (42.92%)	3,284.00 (51.84%)	2,548.00 (39.66%)	
Female	11,879.00 (51.98%)	2,509.00 (47.74%)	2,502.00 (57.08%)	3,187.00 (48.16%)	3,681.00 (60.34%)	
Race						<0.001
Non-Hispanic White	10,215.00 (74.29%)	2,446.00 (76.79%)	2,038.00 (71.91%)	3,046.00 (75.00%)	2,685.00 (71.50%)	
Non-Hispanic Black	5,487.00 (10.63%)	1,502.00 (12.10%)	1,215.00 (11.36%)	1,581.00 (10.55%)	1,189.00 (8.65%)	
Mexican American	5,654.00 (6.86%)	971.00 (4.90%)	1,160.00 (4.88%)	1,545.00 (7.46%)	1,978.00 (9.22%)	
Other	1,116.00 (8.22%)	200.00 (6.21%)	240.00 (11.85%)	299.00 (6.98%)	377.00 (10.63%)	
FIRP						<0.001
0–0.99	4,344.00 (11.12%)	1,235.00 (15.30%)	1,003.00 (11.44%)	1,081.00 (9.53%)	1,025.00 (8.58%)	
≥ 1	16,426.00 (83.93%)	3,556.00 (80.07%)	3,190.00 (81.91%)	4,982.00 (85.76%)	4,698.00 (86.62%)	
Unknown	1,702.00 (4.94%)	328.00 (4.62%)	460.00 (6.65%)	408.00 (4.71%)	506.00 (4.80%)	
Education						<0.001
Below high school	8,034.00 (20.50%)	1,776.00 (22.48%)	2,277.00 (32.16%)	1,756.00 (16.75%)	2,225.00 (18.04%)	
High school	6,361.00 (28.72%)	1,744.00 (34.88%)	1,208.00 (30.12%)	1,940.00 (28.02%)	1,469.00 (21.88%)	
Above high school	7,976.00 (50.56%)	1,577.00 (42.39%)	1,139.00 (37.21%)	2,756.00 (55.07%)	2,504.00 (59.92%)	
Unknown	101.00 (0.23%)	22.00 (0.25%)	29.00 (0.52%)	19.00 (0.16%)	31.00 (0.17%)	
Marital status						<0.001
Married	12,786.00 (60.24%)	2,516.00 (53.82%)	2,693.00 (60.56%)	3,704.00 (61.73%)	3,873.00 (65.24%)	
Other	9,653.00 (39.63%)	2,598.00 (46.02%)	1,952.00 (39.07%)	2,760.00 (38.23%)	2,343.00 (34.64%)	
Unknown	33.00 (0.13%)	5.00 (0.16%)	8.00 (0.37%)	7.00 (0.04%)	13.00 (0.12%)	
BMI (kg/m^2^)						<0.001
< 25	7,984.00 (37.08%)	1,569.00 (29.72%)	1,635.00 (39.87%)	2,143.00 (34.18%)	2,637.00 (48.82%)	
25–30	7,865.00 (33.61%)	1,531.00 (29.14%)	1,712.00 (35.19%)	2,265.00 (35.39%)	2,357.00 (35.17%)	
≥ 30	6,459.00 (28.54%)	1,959.00 (39.95%)	1,286.00 (24.51%)	2,010.00 (29.65%)	1,204.00 (15.55%)	
Unknown	164.00 (0.77%)	60.00 (1.19%)	20.00 (0.43%)	53.00 (0.78%)	31.00 (0.46%)	
Smoking status						<0.001
Never	11,278.00 (48.47%)	1,972.00 (36.81%)	2,191.00 (46.16%)	3,319.00 (50.00%)	3,796.00 (60.87%)	
Former	5,769.00 (25.72%)	1,051.00 (21.18%)	1,416.00 (31.38%)	1,574.00 (25.18%)	1,728.00 (28.92%)	
Current	5,418.00 (25.79%)	2,094.00 (41.97%)	1,046.00 (22.46%)	1,575.00 (24.80%)	703.00 (10.18%)	
Unknown	7.00 (0.03%)	2.00 (0.04%)	0.00 (0.00%)	3.00 (0.02%)	2.00 (0.03%)	
Drinking status						<0.001
Non-drinker	7,452.00 (27.72%)	1,458.00 (25.63%)	1,883.00 (37.48%)	1,910.00 (25.18%)	2,201.00 (29.08%)	
Drinker	11,587.00 (62.43%)	3,042.00 (66.22%)	1,723.00 (44.53%)	3,880.00 (67.47%)	2,942.00 (59.34%)	
Unknown	3,433.00 (9.85%)	619.00 (8.14%)	1,047.00 (17.99%)	681.00 (7.34%)	1,086.00 (11.59%)	
Hypertension						<0.001
No	13,937.00 (66.13%)	3,204.00 (64.21%)	2,531.00 (60.93%)	4,304.00 (67.71%)	3,898.00 (68.57%)	
Yes	8,526.00 (33.82%)	1,915.00 (35.79%)	2,120.00 (39.00%)	2,165.00 (32.24%)	2,326.00 (31.31%)	
Unknown	9.00 (0.05%)	0.00 (0.00%)	2.00 (0.07%)	2.00 (0.05%)	5.00 (0.11%)	
High cholesterol						<0.001
No	15,103.00 (65.21%)	3,901.00 (72.44%)	3,334.00 (68.76%)	4,242.00 (63.09%)	3,626.00 (58.27%)	
Yes	7,360.00 (34.76%)	1,215.00 (27.53%)	1,317.00 (31.20%)	2,226.00 (36.88%)	2,602.00 (41.72%)	
Unknown	9.00 (0.03%)	3.00 (0.03%)	2.00 (0.03%)	3.00 (0.03%)	1.00 (0.00%)	
Diabetes						<0.001
No	18,255.00 (86.85%)	4,242.00 (86.61%)	3,363.00 (76.47%)	5,630.00 (90.60%)	5,020.00 (86.60%)	
Yes	4,217.00 (13.15%)	877.00 (13.39%)	1,290.00 (23.53%)	841.00 (9.40%)	1,209.00 (13.40%)	
CVD						<0.001
No	16,667.00 (81.15%)	3,842.00 (82.00%)	3,038.00 (67.63%)	5,193.00 (86.07%)	4,594.00 (79.42%)	
Yes	5,599.00 (18.27%)	1,230.00 (17.44%)	1,569.00 (31.78%)	1,226.00 (13.44%)	1,574.00 (19.87%)	
Unknown	206.00 (0.57%)	47.00 (0.56%)	46.00 (0.60%)	52.00 (0.49%)	61.00 (0.71%)	
Cancer						0.3
No	21,216.00 (93.46%)	4,851.00 (93.67%)	4,382.00 (92.55%)	6,114.00 (93.63%)	5,869.00 (93.40%)	
Yes	1,242.00 (6.45%)	264.00 (6.17%)	269.00 (7.45%)	352.00 (6.26%)	357.00 (6.54%)	
Unknown	14.00 (0.10%)	4.00 (0.16%)	2.00 (0.01%)	5.00 (0.11%)	3.00 (0.05%)	
α-carotene (ug/dL)	1.10 (0.47, 1.67)	0.00 (−0.03, 0.55)	1.39 (0.96, 1.61)	1.13 (0.74, 1.50)	2.00 (1.65, 2.38)	<0.001
β-carotene (ug/dL)	2.58 (2.08, 3.12)	1.85 (1.59, 2.14)	2.64 (2.30, 3.00)	2.60 (2.30, 2.93)	3.40 (3.09, 3.76)	<0.001
Lycopene (ug/dL)	3.47 (3.04, 3.83)	3.31 (2.94, 3.66)	2.64 (2.30, 2.89)	3.73 (3.43, 4.00)	3.50 (3.14, 3.84)	<0.001
β-cryptoxanthin (ug/dL)	1.95 (1.61, 2.40)	1.43 (1.16, 1.70)	1.79 (1.61, 2.15)	2.08 (1.79, 2.33)	2.58 (2.30, 2.92)	<0.001
Lutein/zeaxanthin (ug/dL)	2.77 (2.48, 3.09)	2.39 (2.15, 2.60)	2.92 (2.71, 3.18)	2.77 (2.54, 3.00)	3.22 (3.00, 3.48)	<0.001

Data were described as medians (interquartile ranges) for continuous variables or weighted % for categorical variables. BMI, body mass index; FIPR, family income to poverty ratio; CVD, cardiovascular diseases.

a Cluster 1, low-level exposure group.

b Cluster 2, low-lycopene exposure group.

c Cluster 3, high-lycopene exposure group.

d Cluster 4, high-level exposure group.

e Differential analyses were conducted using the sampling-weighted analysis of the Wilcoxon rank-sum test for continuous variables, and the chi-squared test with Rao & Scott’s second-order correction for categorical variables.

### 3.2 Individual serum carotenoids and mortality risk

Supplementary Table S3 presents the associations between serum carotenoid concentrations and all-cause mortality risk. In the fully adjusted models, the highest quartiles of the five carotenoids had reduced risks of all-cause mortality compared to the lowest quartile, with HRs (95% CIs) of 0.67 (0.61, 0.75) for α-carotene, 0.71 (0.65, 0.77) for β-carotene, 0.81 (0.72, 0.90) for lycopene, 0.80 (0.73, 0.88) for β-cryptoxanthin, and 0.72 (0.67, 0.77) for lutein/zeaxanthin.

The associations of serum carotenoid concentrations with CVD mortality risk are shown in Supplementary Table S4. In Model 3, the highest quartiles of serum α-carotene and β-carotene were associated with reduced risks of CVD mortality compared to their respective lowest quartiles, with HRs (95% CIs) of 0.71 (0.58, 0.88) and 0.76 (0.63, 0.90), respectively. For lycopene, the other quartile groups showed statistically significant HRs ranging from 0.86 to 0.66 for CVD mortality compared to the lowest quartile. Additionally, participants in the second and third quartiles of lutein/zeaxanthin had decreased risks of CVD mortality, with HRs (95% CIs) of 0.82 (0.71, 0.94) and 0.74 (0.63, 0.88), respectively, compared to those in the lowest quartile. However, there was no significant association between β-cryptoxanthin and CVD mortality risk in Model 3.

Supplementary Table S5 displays the relationships between serum carotenoid concentrations and cancer mortality risk. After adjusting for all covariates, the highest quartiles of α-carotene, β-carotene, lycopene, β-cryptoxanthin, and lutein/zeaxanthin were significantly associated with lower risks of cancer mortality compared to their respective lowest quartiles, with HRs (95% CIs) of 0.74 (0.60, 0.92), 0.75 (0.63, 0.90), 0.50 (0.39, 0.63), 0.60 (0.50, 0.73), and 0.82 (0.70, 0.95), respectively.

### 3.3 Exposure-response of individual carotenoids on the risk of mortality risk

**Figure 5:**
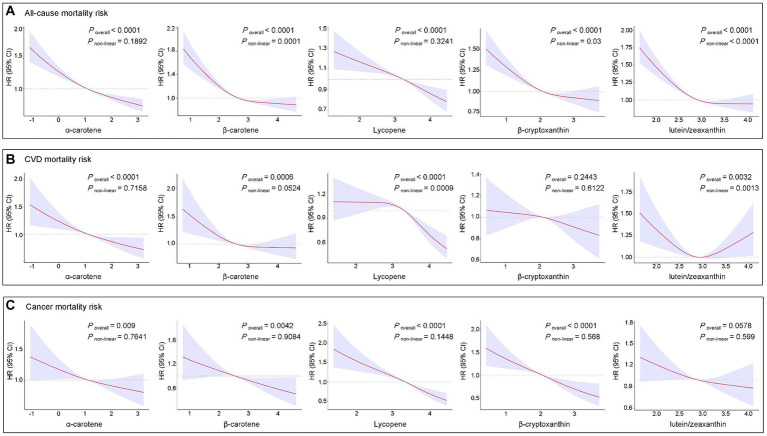
Restricted cubic spline analyses of the association of serum carotenoids with all-cause **(A)**, CVD **(B)**, and cancer **(C)** mortality. Adjusted for age (continuous), sex, race, FIPR, education level, BMI, marital status, smoking status, alcohol consumption, histories of hypertension, high cholesterol, diabetes, CVD, and cancer. BMI, body mass index; CI, confidence interval; CVD, cardiovascular diseases; FIPR, family income to poverty ratio; HR, hazard ratio.

Figure 5 illustrates the exposure-response relationships between circulating levels of each studied carotenoid and mortality risk. After adjusting for all covariates, nonlinear associations were observed for β-carotene (*P*_overall_ < 0.0001 and *P*_non-linear_ = 0.0001), β-cryptoxanthin (*P*_overall_ < 0.0001 and *P*_non-linear_ = 0.03), and lutein/zeaxanthin (*P*_overall_ < 0.0001 and *P*_non-linear_ < 0.0001) with all-cause mortality. Additionally, nonlinear exposure-response relationships were observed between lycopene (*P*_overall_ < 0.0001 and *P*_non-linear_ = 0.0009) and lutein/zeaxanthin (*P*_overall_ = 0.0032 and *P*_non-linear_ = 0.0013) with CVD mortality. However, no significant nonlinear associations were observed between the five studied carotenoids and cancer mortality.

### 3.4 Co-exposure of multiple carotenoids and mortality risks

The associations between carotenoid co-exposure patterns and mortality risks are presented in Table 2. In the fully adjusted model, participants in the low-lycopene, high-lycopene, and high-level exposure groups exhibited lower all-cause mortality risks compared to those in the low-level exposure group, with HRs (95% CIs) of 0.79 (0.72, 0.87), 0.75 (0.67, 0.84), and 0.67 (0.61, 0.74), respectively. Regarding CVD mortality, the high-lycopene exposure group demonstrated a 27% reduced risk (HR: 0.73, 95% CI: 0.61–0.86) compared to the low-level exposure group. Similarly, the high-level exposure group noted a 21% reduced risk (HR: 0.79, 95% CI: 0.67–0.93). Furthermore, compared to the low-level exposure group, the high-lycopene and high-level exposure groups exhibited 30% and 35% lower risks of cancer mortality, with HRs (95% CIs) of 0.70 (0.57, 0.86) and 0.65 (0.54, 0.79), respectively.

**Table 2:** Association between multi-carotenoid co-exposure clusters and mortality risk in NHANES III and NHANES 2003–2006^e^.

Model	Cluster 1^a^	Cluster 2^b^	Cluster 3^c^	Cluster 4^d^
All-cause mortality				
No. deaths/total	1552/5119	2610/4653	1461/6471	2278/6229
Model 1 HR (95% CI)	1.00 (reference)	**0.75 (0.69, 0.82)**	**0.64 (0.57, 0.71)**	**0.52 (0.47, 0.57)**
Model 2 HR (95% CI)	1.00 (reference)	**0.80 (0.73, 0.87)**	**0.73 (0.65, 0.81)**	**0.65 (0.60, 0.71)**
Model 3 HR (95% CI)	1.00 (reference)	**0.79 (0.72, 0.87)**	**0.75 (0.67, 0.84)**	**0.67 (0.61, 0.74)**
CVD mortality				
No. deaths/total	473/4040	997/3040	480/5490	921/4872
Model 1 HR (95% CI)	1.00 (reference)	0.89 (0.75, 1.05)	**0.60 (0.51, 0.72)**	**0.59 (0.50, 0.70)**
Model 2 HR (95% CI)	1.00 (reference)	0.91 (0.77, 1.08)	**0.70 (0.60, 0.81)**	**0.75 (0.65, 0.87)**
Model 3 HR (95% CI)	1.00 (reference)	0.86 (0.73, 1.02)	**0.73 (0.61, 0.86)**	**0.79 (0.67, 0.93)**
Cancer mortality				
No. deaths/total	374/3941	570/2613	349/5359	424/4375
Model 1 HR (95% CI)	1.00 (reference)	0.95 (0.75, 1.21)	**0.57 (0.47, 0.70)**	**0.46 (0.38, 0.55)**
Model 2 HR (95% CI)	1.00 (reference)	1.03 (0.81, 1.32)	**0.68 (0.56, 0.84)**	**0.63 (0.52, 0.76)**
Model 3 HR (95% CI)	1.00 (reference)	1.00 (0.78, 1.27)	**0.70 (0.57, 0.86)**	**0.65 (0.54, 0.79)**

Bold indicates statistical significance. Model 1 was adjusted for age (continuous), sex, race, and FIPR. Model 2 was further adjusted for education level, BMI, marital status, smoking status, and alcohol consumption. Model 3 was additionally adjusted for histories of hypertension, high cholesterol, diabetes, CVD, and cancer. BMI, body mass index; CI, confidence interval; CVD, cardiovascular diseases; FIPR, family income to poverty ratio; HR, hazard ratio; NHANES, National Health and Nutrition Examination Survey.

a Cluster 1, low-level exposure group.

b Cluster 2, low-lycopene exposure group.

c Cluster 3, high-lycopene exposure group.

d Cluster 4, high-level exposure group.

e All estimates were weight-adjusted using NHANES-specified sampling weights.

### 3.5 Sensitivity analysis

In the sensitivity analyses, Cox proportional hazards analysis was conducted after excluding participants with missing covariate values. The results were consistent with those of the primary analysis (Supplementary Table S6). Additionally, participants who died within the first 2 years of follow-up were excluded, and the results remained consistent with the primary analysis (Supplementary Table S7).

## 4 Discussion

In this large prospective cohort study, we observed that higher serum levels of most types of carotenoids were associated with lower all-cause, CVD, and cancer mortality risk. Considering the complex interactions among serum carotenoids, our study further suggested that simultaneous exposure to elevated levels of carotenoid mixtures was associated with a reduced mortality risk. Several sensitivity analyses confirmed the robustness of our findings.

The associations between carotenoids and mortality risk have recently garnered increasing attention (15, 17, 19–22, 28–33). Despite inconsistent findings, most studies revealed inverse associations between circulating carotenoid levels and mortality risk (15, 19–22, 29–33). For example, a pooled analysis of 69 prospective studies reported that high circulating levels of α-carotene, β-carotene, and total carotenoids were inversely associated with mortality risk (34). Similarly, negative associations between serum levels of most carotenoid types and mortality risk were observed in the Japanese population (15). Furthermore, significant inverse associations were observed between dietary total carotene intake and the risk of CVD mortality, but not cancer mortality, in Chinese adults (35). Our findings confirm and expand upon a previous study involving 13,293 participants from NHANES III, which assessed the influence of serum carotenoids on mortality risk with follow-up until December 31, 2006 (14). The results indicated that low serum levels of α-carotene and lycopene were associated with increased risks of all-cause mortality. With a larger sample size and more extended follow-up period than the previous study (14), we observed that, in addition to α-carotene and lycopene, low serum levels of β-carotene, β-cryptoxanthin, and lutein/zeaxanthin were also associated with increased risks of all-cause mortality. Despite indications from some interventional studies that supplementation with β-carotene may have null or harmful effects on mortality outcomes (16, 36), higher circulating carotenoid levels did not correlate with elevated mortality risk in our study.

Humans are often exposed to multiple carotenoids simultaneously, leading to potential interactions among them (9). For example, the interaction between lycopene and lutein/zeaxanthin has been significantly associated with all-cause mortality (14). To further investigate this, we examined the association between co-exposure patterns of carotenoids and mortality risk using an unsupervised clustering model. Our findings indicate that individuals in the high-level exposure group, characterized by elevated co-exposure to multiple carotenoids, had a decreased risk of all-cause, CVD, and cancer mortality. Furthermore, individuals in the high-lycopene exposure group, with moderate levels of α-carotene, β-carotene, β-cryptoxanthin, and lutein/zeaxanthin, also showed a reduced risk of these mortality outcomes. These results underscore the potential protective effects of carotenoids against various fatal diseases. Moreover, the specific finding related to the high-lycopene exposure group suggests that lycopene, even when accompanied by moderate levels of other carotenoids, significantly reduces mortality risks, especially for CVD mortality. Our results were consistent with previous research showing the beneficial effects of carotenoids, particularly lycopene, on cardiovascular health (37), and the combined protective effect of lycopene with other carotenoids may be due to their synergistic actions (9). Overall, our findings provided evidence supporting the potential health benefits associated with higher levels of carotenoid intake, particularly lycopene (38). Nevertheless, further research, including randomized controlled trials, is needed to confirm the protective effects of carotenoids against mortality from different causes.

Carotenoids play a crucial role in health outcomes, and the mechanisms underlying their effects warrant further exploration. Carotenoids have numerous essential biological functions found in plants, algae, and certain bacteria, including antioxidation, anticancer, anti-inflammatory, and immunomodulatory effects (6, 9, 39). For instance, carotenoids function as antioxidants by scavenging and neutralizing reactive oxygen species (ROS) and free radicals within the body (40, 41). This process safeguards cells against oxidative damage, reducing the risk of chronic diseases such as Parkinson’s, diabetes, and CVD (42). Additionally, α-carotene and lycopene have been shown to inhibit the migration and invasion of various cancer cells (43–45). Furthermore, growing evidence supports that β-carotene can inhibit the expression of pro-inflammatory mediators, including NO, prostaglandin E2 (PGE2), inducible iNOS, COX-2, TNF-α, and IL-1β, by acting as an inhibitor of NF-κB activation (46). Both *in vitro* and *in vivo* studies have shown that β-cryptoxanthin may have beneficial effects on health and the prevention of immune-related diseases by elevating CD4+ lymphocytes and serum levels of immunoglobulins IgG, IgM, and IgA in mammals (47, 48). Moreover, several studies have demonstrated that lutein and zeaxanthin can suppress the expression of inflammatory mediators in immune cells and reduce inflammation in conditions such as age-related macular degeneration and neurodegenerative diseases (49–51). While these mechanisms offer insight into how carotenoids decrease mortality risk, their effects are likely complex, potentially involving interactions with other nutrients and biological processes. For example, Stahl et al. discovered that combinations of carotenoids are more effective than individual compounds in preventing oxidative damage (52). These effects may stem from the distinct physicochemical properties and distribution of carotenoids within biomembranes (53). Similarly, a recent study demonstrated that combining β-carotene and lycopene has a stronger effect on the expression of genes involved in antioxidant defense than each carotenoid alone (54). However, research on the underlying mechanisms of potential interactions between different carotenoids is still relatively scarce. Therefore, further study is necessary to fully understand the mechanisms behind the complex interactions between different carotenoids and their potential synergistic or antagonistic effects on health.

The strengths of our study included its prospective design, large sample size, long-term follow-up, comprehensive data on potential confounders, and the utilization of multiple carotenoid co-exposure patterns derived from unsupervised machine learning methods. Nonetheless, there are still several limitations. First, a single measurement of serum carotenoids at baseline may not reflect long-term exposures. Future studies should explore whether and how fluctuations in carotenoid levels affect mortality risk. Second, all participants in this study were adults from the United States; therefore, caution should be taken when broadly applying our results to other populations. Third, the clustering results indicate only the levels of carotenoids, not their categories; thus, the findings cannot reflect the specific contribution of each carotenoid. Lastly, the k-means clustering method is sensitive to outliers (55). To mitigate this issue, we excluded participants with the most extreme 1% values for the five serum carotenoids and normalized the data.

## 5 Conclusion

Our study suggested that co-exposure to multiple serum carotenoids was associated with reduced risks of all-cause, CVD, and cancer mortality. These findings implied potential health benefits from diets rich in diverse carotenoids. However, further research is necessary to understand the underlying biological mechanisms and to confirm these associations across different populations. Public health strategies encouraging the consumption of carotenoid-rich foods could contribute to improved health outcomes.

## Data availability statement

The original contributions presented in the study are included in the article/Supplementary material, further inquiries can be directed to the corresponding authors.

## Ethics statement

The studies involving humans were approved by National Center for Health Statistics Ethics Review Board. The studies were conducted in accordance with the local legislation and institutional requirements. Written informed consent for participation in this study was provided by the participants' legal guardians/next of kin.

## Author contributions

QH: Conceptualization, Formal analysis, Methodology, Software, Writing – original draft, Data curation, Writing – review & editing. CY: Methodology, Writing – review & editing. ZL: Conceptualization, Funding acquisition, Methodology, Writing – review & editing. XW: Conceptualization, Formal analysis, Methodology, Validation, Writing – review & editing.

## Footnotes

^1^https://www.cdc.gov/nchs/data-linkage/mortality-public.htm
