# Supplementary material for: Associations of multiple carotenoid co-exposure with all-cause and cause-specific mortality in US adults: a prospective cohort study
Source: Front Nutr. 2024 Aug 7;11:1415537. doi: 10.3389/fnut.2024.1415537 (PMC11335626; doi:10.3389/fnut.2024.1415537)
Supplement: Supplementary file 1 [file Table_1.DOCX]

**Supplementary tables**

**Table S1** Distributions of standardized levels of the serum carotenoids.

| **Variables** | **Min** | **25^th^ percentile** | **Median** | **75^th^ percentile** | **Max** |
| --- | --- | --- | --- | --- | --- |
| α-carotene | -2.80 | -0.71 | -0.01 | 0.62 | 2.58 |
| β-carotene | -2.70 | -0.79 | 0.00 | 0.69 | 2.81 |
| Lycopene | -3.35 | -0.65 | 0.08 | 0.72 | 2.11 |
| β-cryptoxanthin | -2.92 | -0.74 | -0.07 | 0.73 | 2.69 |
| Lutein/zeaxanthin | -2.81 | -0.72 | -0.04 | 0.68 | 2.60 |

**Table S2** Centres of the four clusters of the five serum carotenoids.

| **Variables** | **Cluster 1^a^** | **Cluster 2^b^** | **Cluster 3^c^** | **Cluster 4^d^** |
| --- | --- | --- | --- | --- |
| α-carotene | -1.16 | 0.02 | -0.05 | 0.99 |
| β-carotene | -1.11 | 0.01 | -0.09 | 1.00 |
| Lycopene | -0.14 | -1.15 | 0.72 | 0.23 |
| β-cryptoxanthin | -0.99 | -0.25 | 0.04 | 0.96 |
| Lutein/zeaxanthin | -0.96 | 0.14 | -0.16 | 0.85 |

^a^ Cluster 1, low-level exposure group. ^b^ Cluster 2, low-lycopene exposure group. ^c^ Cluster 3, high-lycopene exposure group. ^d^ Cluster 4, high-level exposure group.

**Table S3** Association between serum carotenoid concentrations and all-cause mortality risk in NHANES III and NHANES 2003–2006^*^.

| **Model** | **Quartile 1** | **Quartile 2** | **Quartile 3** | **Quartile 4** | ***P*_trend_** | **Per 1-SD increment** |
| --- | --- | --- | --- | --- | --- | --- |
| **α-carotene** |  |  |  |  |  |  |
| No. deaths/total | 1766/5465 | 1912/5756 | 2214/5778 | 2009/5473 |  |  |
| Model 1 HR (95% CI) | 1.00 (reference) | **0.74 (0.66, 0.83)** | **0.64 (0.58, 0.72)** | **0.53 (0.48, 0.58)** | **<0.001** | **0.77 (0.74, 0.80)** |
| Model 2 HR (95% CI) | 1.00 (reference) | **0.79 (0.71, 0.88)** | **0.75 (0.67, 0.83)** | **0.66 (0.60, 0.73)** | **<0.001** | **0.84 (0.81, 0.87)** |
| Model 3 HR (95% CI) | 1.00 (reference) | **0.80 (0.71, 0.90)** | **0.76 (0.68, 0.85)** | **0.67 (0.61, 0.75)** | **<0.001** | **0.85 (0.81, 0.88)** |
| **β-carotene** |  |  |  |  |  |  |
| No. deaths/total | 1605/5628 | 1633/5159 | 2123/5896 | 2540/5789 |  |  |
| Model 1 HR (95% CI) | 1.00 (reference) | **0.78 (0.71, 0.86)** | **0.64 (0.58, 0.70)** | **0.56 (0.51, 0.61)** | **<0.001** | **0.80 (0.77, 0.83)** |
| Model 2 HR (95% CI) | 1.00 (reference) | **0.82 (0.75, 0.90)** | **0.72 (0.65, 0.78)** | **0.67 (0.61, 0.73)** | **<0.001** | **0.86 (0.83, 0.88)** |
| Model 3 HR (95% CI) | 1.00 (reference) | **0.84 (0.76, 0.92)** | **0.74 (0.68, 0.81)** | **0.71 (0.65, 0.77)** | **<0.001** | **0.88 (0.85, 0.90)** |
| **Lycopene** |  |  |  |  |  |  |
| No. deaths/total | 4585/8978 | 1849/6038 | 982/4245 | 485/3211 |  |  |
| Model 1 HR (95% CI) | 1.00 (reference) | **0.79 (0.73, 0.86)** | **0.74 (0.68, 0.82)** | **0.65 (0.57, 0.73)** | **<0.001** | **0.86 (0.83, 0.89)** |
| Model 2 HR (95% CI) | 1.00 (reference) | **0.84 (0.77, 0.91)** | **0.81 (0.73, 0.89)** | **0.74 (0.66, 0.82)** | **<0.001** | **0.89 (0.87, 0.93)** |
| Model 3 HR (95% CI) | 1.00 (reference) | **0.86 (0.80, 0.94)** | **0.85 (0.77, 0.93)** | **0.81 (0.72, 0.90)** | **<0.001** | **0.92 (0.89, 0.95)** |
| **β-cryptoxanthin** |  |  |  |  |  |  |
| No. deaths/total | 2294/5356 | 1546/4559 | 1868/5578 | 2193/6979 |  |  |
| Model 1 HR (95% CI) | 1.00 (reference) | **0.82 (0.75, 0.91)** | **0.72 (0.66, 0.79)** | **0.62 (0.55, 0.68)** | **<0.001** | **0.82 (0.79, 0.85)** |
| Model 2 HR (95% CI) | 1.00 (reference) | **0.90 (0.82, 0.98)** | **0.82 (0.75, 0.91)** | **0.77 (0.70, 0.86)** | **<0.001** | **0.89 (0.86, 0.92)** |
| Model 3 HR (95% CI) | 1.00 (reference) | **0.92 (0.84, 1.00)** | **0.85 (0.77, 0.94)** | **0.80 (0.73, 0.88)** | **<0.001** | **0.90 (0.87, 0.94)** |
| **Lutein/zeaxanthin** |  |  |  |  |  |  |
| No. deaths/total | 1199/3815 | 1663/5180 | 1920/5865 | 3119/7612 |  |  |
| Model 1 HR (95% CI) | 1.00 (reference) | **0.79 (0.72, 0.87)** | **0.66 (0.60, 0.72)** | **0.62 (0.58, 0.67)** | **<0.001** | **0.84 (0.82, 0.86)** |
| Model 2 HR (95% CI) | 1.00 (reference) | **0.82 (0.76, 0.88)** | **0.71 (0.64, 0.78)** | **0.71 (0.67, 0.76)** | **<0.001** | **0.88 (0.86, 0.91)** |
| Model 3 HR (95% CI) | 1.00 (reference) | **0.83 (0.77, 0.89)** | **0.72 (0.65, 0.79)** | **0.72 (0.67, 0.77)** | **<0.001** | **0.89 (0.87, 0.91)** |

Bold indicates statistical significance. ^*^All estimates were weight adjusted using NHANES-specified sampling weights. Model 1 was adjusted for age (continuous), sex, race, and FIPR. Model 2 was further adjusted for education level, BMI, marital status, smoking status, and alcohol consumption. Model 3 was additionally adjusted for histories of hypertension, high cholesterol, diabetes, CVD, and cancer. BMI, body mass index; CI, confidence interval; CVD, cardiovascular diseases; FIPR, family income to poverty ratio; HR, hazard ratio; NHANES, National Health and Nutrition Examination Survey; SD, standard deviation.

**Table S4** Association between serum carotenoid concentrations and CVD mortality risk in NHANES III and NHANES 2003–2006^*^.

| **Model** | **Quartile 1** | **Quartile 2** | **Quartile 3** | **Quartile 4** | ***P*_trend_** | **Per 1-SD increment** |
| --- | --- | --- | --- | --- | --- | --- |
| **α-carotene** |  |  |  |  |  |  |
| No. deaths/total | 565/4264 | 677/4521 | 824/4388 | 805/4269 |  |  |
| Model 1 HR (95% CI) | 1.00 (reference) | **0.76 (0.60, 0.95)** | **0.68 (0.55, 0.82)** | **0.56 (0.46, 0.68)** | **<0.001** | **0.77 (0.72, 0.83)** |
| Model 2 HR (95% CI) | 1.00 (reference) | 0.80 (0.64, 1.00) | **0.78 (0.65, 0.95)** | **0.70 (0.58, 0.85)** | **0.001** | **0.84 (0.78, 0.91)** |
| Model 3 HR (95% CI) | 1.00 (reference) | 0.81 (0.64, 1.02) | **0.81 (0.67, 0.99)** | **0.71 (0.58, 0.88)** | **0.003** | **0.85 (0.79, 0.92)** |
| **β-carotene** |  |  |  |  |  |  |
| No. deaths/total | 487/4510 | 564/4090 | 790/4563 | 1030/4279 |  |  |
| Model 1 HR (95% CI) | 1.00 (reference) | **0.83 (0.69, 1.00)** | **0.70 (0.58, 0.84)** | **0.58 (0.49, 0.69)** | **<0.001** | **0.82 (0.77, 0.87)** |
| Model 2 HR (95% CI) | 1.00 (reference) | 0.86 (0.73, 1.02) | **0.78 (0.65, 0.94)** | **0.69 (0.58, 0.83)** | **<0.001** | **0.87 (0.82, 0.93)** |
| Model 3 HR (95% CI) | 1.00 (reference) | 0.87 (0.72, 1.04) | **0.82 (0.68, 0.98)** | **0.76 (0.63, 0.90)** | **0.002** | **0.91 (0.86, 0.97)** |
| **Lycopene** |  |  |  |  |  |  |
| No. deaths/total | 1708/6101 | 640/4829 | 349/3612 | 174/2900 |  |  |
| Model 1 HR (95% CI) | 1.00 (reference) | **0.74 (0.65, 0.83)** | **0.62 (0.54, 0.71)** | **0.47 (0.38, 0.58)** | **<0.001** | **0.80 (0.75, 0.85)** |
| Model 2 HR (95% CI) | 1.00 (reference) | **0.80 (0.71, 0.91)** | **0.69 (0.60, 0.80)** | **0.56 (0.45, 0.69)** | **<0.001** | **0.85 (0.79, 0.90)** |
| Model 3 HR (95% CI) | 1.00 (reference) | **0.86 (0.77, 0.97)** | **0.76 (0.66, 0.87)** | **0.66 (0.52, 0.84)** | **<0.001** | **0.89 (0.83, 0.95)** |
| **β-cryptoxanthin** |  |  |  |  |  |  |
| No. deaths/total | 794/3856 | 549/3562 | 676/4386 | 852/5638 |  |  |
| Model 1 HR (95% CI) | 1.00 (reference) | 0.95 (0.75, 1.19) | **0.79 (0.68, 0.92)** | **0.71 (0.58, 0.86)** | **<0.001** | **0.87 (0.82, 0.93)** |
| Model 2 HR (95% CI) | 1.00 (reference) | 1.04 (0.84, 1.29) | 0.91 (0.78, 1.05) | 0.89 (0.74, 1.07) | 0.089 | 0.94 (0.89, 1.00) |
| Model 3 HR (95% CI) | 1.00 (reference) | 1.13 (0.92, 1.39) | 0.94 (0.81, 1.09) | 0.94 (0.79, 1.12) | 0.200 | 0.96 (0.90, 1.02) |
| **Lutein/zeaxanthin** |  |  |  |  |  |  |
| No. deaths/total | 402/3018 | 540/4057 | 689/4634 | 1240/5733 |  |  |
| Model 1 HR (95% CI) | 1.00 (reference) | **0.75 (0.63, 0.90)** | **0.66 (0.57, 0.77)** | **0.73 (0.62, 0.86)** | **<0.001** | **0.90 (0.85, 0.96)** |
| Model 2 HR (95% CI) | 1.00 (reference) | **0.80 (0.68, 0.93)** | **0.72 (0.62, 0.84)** | 0.86 (0.74, 1.00) | 0.100 | 0.96 (0.90, 1.02) |
| Model 3 HR (95% CI) | 1.00 (reference) | **0.82 (0.71, 0.94)** | **0.74 (0.63, 0.88)** | 0.87 (0.73, 1.04) | 0.200 | 0.96 (0.90, 1.03) |

Bold indicates statistical significance. ^*^All estimates were weight adjusted using NHANES-specified sampling weights. Model 1 was adjusted for age (continuous), sex, race, and FIPR. Model 2 was further adjusted for education level, BMI, marital status, smoking status, and alcohol consumption. Model 3 was additionally adjusted for histories of hypertension, high cholesterol, diabetes, CVD, and cancer. BMI, body mass index; CI, confidence interval; CVD, cardiovascular diseases; FIPR, family income to poverty ratio; HR, hazard ratio; NHANES, National Health and Nutrition Examination Survey; SD, standard deviation.

**Table S5** Association between serum carotenoid concentrations and cancer mortality risk in NHANES III and NHANES 2003–2006^*^.

| **Model** | **Quartile 1** | **Quartile 2** | **Quartile 3** | **Quartile 4** | ***P*_trend_** | **Per 1-SD increment** |
| --- | --- | --- | --- | --- | --- | --- |
| **α-carotene** |  |  |  |  |  |  |
| No. deaths/total | 420/4119 | 420/4264 | 494/4058 | 383/3847 |  |  |
| Model 1 HR (95% CI) | 1.00 (reference) | **0.71 (0.58, 0.88)** | **0.72 (0.58, 0.89)** | **0.51 (0.41, 0.62)** | **<0.001** | **0.78 (0.72, 0.83)** |
| Model 2 HR (95% CI) | 1.00 (reference) | **0.78 (0.63, 0.96)** | 0.88 (0.71, 1.09) | **0.71 (0.57, 0.89)** | **0.012** | **0.88 (0.82, 0.96)** |
| Model 3 HR (95% CI) | 1.00 (reference) | **0.79 (0.64, 0.98)** | 0.91 (0.74, 1.13) | **0.74 (0.60, 0.92)** | **0.030** | **0.90 (0.83, 0.97)** |
| **β-carotene** |  |  |  |  |  |  |
| No. deaths/total | 397/4420 | 375/3091 | 469/4242 | 476/3725 |  |  |
| Model 1 HR (95% CI) | 1.00 (reference) | 0.89 (0.71, 1.12) | **0.69 (0.58, 0.82)** | **0.55 (0.46, 0.66)** | **<0.001** | **0.79 (0.74, 0.84)** |
| Model 2 HR (95% CI) | 1.00 (reference) | 0.92 (0.74, 1.16) | **0.82 (0.68, 0.98)** | **0.70 (0.58, 0.85)** | **<0.001** | **0.87 (0.81, 0.93)** |
| Model 3 HR (95% CI) | 1.00 (reference) | 0.95 (0.77, 1.17) | **0.83 (0.70, 0.99)** | **0.75 (0.63, 0.90)** | **<0.001** | **0.89 (0.83, 0.95)** |
| **Lycopene** |  |  |  |  |  |  |
| No. deaths/total | 964/5357 | 413/4602 | 231/3494 | 109/2835 |  |  |
| Model 1 HR (95% CI) | 1.00 (reference) | **0.57 (0.48, 0.67)** | **0.54 (0.44, 0.66)** | **0.38 (0.30, 0.48)** | **<0.001** | **0.73 (0.68, 0.78)** |
| Model 2 HR (95% CI) | 1.00 (reference) | **0.60 (0.51, 0.71)** | **0.61 (0.50, 0.74)** | **0.45 (0.36, 0.57)** | **<0.001** | **0.77 (0.72, 0.83)** |
| Model 3 HR (95% CI) | 1.00 (reference) | **0.62 (0.52, 0.73)** | **0.64 (0.51, 0.79)** | **0.50 (0.39, 0.63)** | **<0.001** | **0.80 (0.74, 0.86)** |
| **β-cryptoxanthin** |  |  |  |  |  |  |
| No. deaths/total | 554/3616 | 365/3378 | 396/4106 | 402/5188 |  |  |
| Model 1 HR (95% CI) | 1.00 (reference) | **0.80 (0.65, 0.97)** | **0.59 (0.48, 0.73)** | **0.43 (0.35, 0.52)** | **<0.001** | **0.73 (0.68, 0.77)** |
| Model 2 HR (95% CI) | 1.00 (reference) | 0.88 (0.73, 1.05) | **0.70 (0.57, 0.86)** | **0.57 (0.47, 0.69)** | **<0.001** | **0.81 (0.76, 0.86)** |
| Model 3 HR (95% CI) | 1.00 (reference) | 0.89 (0.73, 1.07) | **0.71 (0.58, 0.88)** | **0.60 (0.50, 0.73)** | **<0.001** | **0.83 (0.78, 0.88)** |
| **Lutein/zeaxanthin** |  |  |  |  |  |  |
| No. deaths/total | 266/2882 | 385/3902 | 433/4378 | 633/5126 |  |  |
| Model 1 HR (95% CI) | 1.00 (reference) | 0.88 (0.70, 1.09) | **0.62 (0.49, 0.79)** | **0.65 (0.54, 0.78)** | **<0.001** | **0.85 (0.80, 0.90)** |
| Model 2 HR (95% CI) | 1.00 (reference) | 0.93 (0.76, 1.14) | **0.68 (0.54, 0.87)** | **0.80 (0.68, 0.94)** | **<0.001** | **0.92 (0.87, 0.97)** |
| Model 3 HR (95% CI) | 1.00 (reference) | 0.96 (0.79, 1.17) | **0.70 (0.55, 0.89)** | **0.82 (0.70, 0.95)** | **0.001** | **0.93 (0.88, 0.98)** |

Bold indicates statistical significance. ^*^All estimates were weight adjusted using NHANES-specified sampling weights. Model 1 was adjusted for age (continuous), sex, race, and FIPR. Model 2 was further adjusted for education level, BMI, marital status, smoking status, and alcohol consumption. Model 3 was additionally adjusted for histories of hypertension, high cholesterol, diabetes, CVD, and cancer. BMI, body mass index; CI, confidence interval; CVD, cardiovascular diseases; FIPR, family income to poverty ratio; HR, hazard ratio; NHANES, National Health and Nutrition Examination Survey; SD, standard deviation.

**Table S6** Association between multi-carotenoid co-exposure clusters and mortality risk after excluding participants with missing values for covariates^e^.

| **Model** | **Cluster 1^a^** | **Cluster 2^b^** | **Cluster 3^c^** | **Cluster 4^d^** |
| --- | --- | --- | --- | --- |
| **All-cause mortality** |  |  |  |  |
| No. deaths/total | 1199/4126 | 1749/3223 | 1180/5367 | 1589/4704 |
| Model 1 HR (95% CI) | 1.00 (reference) | **0.80 (0.72, 0.89)** | **0.65 (0.58, 0.73)** | **0.51 (0.46, 0.57)** |
| Model 2 HR (95% CI) | 1.00 (reference) | **0.84 (0.75, 0.94)** | **0.75 (0.67, 0.84)** | **0.65 (0.59, 0.72)** |
| Model 3 HR (95% CI) | 1.00 (reference) | **0.83 (0.74, 0.93)** | **0.77 (0.68, 0.87)** | **0.67 (0.60, 0.75)** |
| **CVD mortality** |  |  |  |  |
| No. deaths/total | 356/3283 | 641/2115 | 394/4581 | 628/3743 |
| Model 1 HR (95% CI) | 1.00 (reference) | 0.92 (0.77, 1.11) | **0.62 (0.52, 0.74)** | **0.55 (0.45, 0.66)** |
| Model 2 HR (95% CI) | 1.00 (reference) | 0.96 (0.79, 1.17) | **0.73 (0.62, 0.86)** | **0.72 (0.61, 0.87)** |
| Model 3 HR (95% CI) | 1.00 (reference) | 0.90 (0.74, 1.08) | **0.76 (0.64, 0.90)** | **0.77 (0.63, 0.94)** |
| **Cancer mortality** |  |  |  |  |
| No. deaths/total | 311/3238 | 409/1883 | 292/4479 | 315/3430 |
| Model 1 HR (95% CI) | 1.00 (reference) | 1.03 (0.79, 1.34) | **0.59 (0.48, 0.72)** | **0.45 (0.36, 0.55)** |
| Model 2 HR (95% CI) | 1.00 (reference) | 1.03 (0.80, 1.33) | **0.62 (0.50, 0.76)** | **0.49 (0.39, 0.60)** |
| Model 3 HR (95% CI) | 1.00 (reference) | 1.06 (0.81, 1.40) | **0.71 (0.57, 0.88)** | **0.63 (0.51, 0.78)** |

Bold indicates statistical significance. ^a^ Cluster 1, low-level exposure group. ^b^ Cluster 2, low-lycopene exposure group. ^c^ Cluster 3, high-lycopene exposure group. ^d^ Cluster 4, high-level exposure group. ^e^ In this analysis, a total of 17,546 participants were included, and all estimates were weight adjusted using NHANES-specified sampling weights. Model 1 was adjusted for age (continuous), sex,, race, and FIPR. Model 2 was further adjusted for education level, BMI, marital status, smoking status, and alcohol consumption. Model 3 was additionally adjusted for histories of hypertension, high cholesterol, diabetes, CVD, and cancer. BMI, body mass index; CI, confidence interval; CVD, cardiovascular diseases; FIPR, family income to poverty ratio; HR, hazard ratio; NHANES, National Health and Nutrition Examination Survey.

**Table S7** Association between multi-carotenoid co-exposure clusters and mortality risk after excluding participants who died within the first 2 years of follow-up^e^.

| **Model** | **Cluster 1^a^** | **Cluster 2^b^** | **Cluster 3^c^** | **Cluster 4^d^** |
| --- | --- | --- | --- | --- |
| **All-cause mortality** |  |  |  |  |
| No. deaths/total | 1445/5012 | 2401/4444 | 1386/6396 | 2172/6123 |
| Model 1 | 1.00 (reference) | **0.72 (0.66, 0.79)** | **0.64 (0.58, 0.71)** | **0.52 (0.47, 0.57)** |
| Model 2 | 1.00 (reference) | **0.77 (0.71, 0.85)** | **0.73 (0.66, 0.82)** | **0.65 (0.59, 0.72)** |
| Model 3 | 1.00 (reference) | **0.76 (0.69, 0.84)** | **0.76 (0.68, 0.85)** | **0.67 (0.61, 0.74)** |
| **CVD mortality** |  |  |  |  |
| No. deaths/total | 438/4005 | 906/2949 | 455/5465 | 864/4815 |
| Model 1 | 1.00 (reference) | **0.84 (0.71, 0.99)** | **0.62 (0.52, 0.73)** | **0.58 (0.49, 0.69)** |
| Model 2 | 1.00 (reference) | 0.85 (0.71, 1.01) | **0.71 (0.60, 0.83)** | **0.73 (0.62, 0.86)** |
| Model 3 | 1.00 (reference) | **0.80 (0.67, 0.96)** | **0.75 (0.62, 0.89)** | **0.77 (0.65, 0.92)** |
| **Cancer mortality** |  |  |  |  |
| No. deaths/total | 345/3912 | 524/2567 | 335/5345 | 415/4366 |
| Model 1 | 1.00 (reference) | 0.94 (0.73, 1.20) | **0.59 (0.48, 0.72)** | **0.48 (0.39, 0.58)** |
| Model 2 | 1.00 (reference) | 1.02 (0.79, 1.32) | **0.70 (0.57, 0.87)** | **0.66 (0.54, 0.81)** |
| Model 3 | 1.00 (reference) | 0.99 (0.76, 1.28) | **0.72 (0.58, 0.89)** | **0.68 (0.56, 0.83)** |

Bold indicates statistical significance. ^a^ Cluster 1, low-level exposure group. ^b^ Cluster 2, low-lycopene exposure group. ^c^ Cluster 3, high-lycopene exposure group. ^d^ Cluster 4, high-level exposure group. ^e^ In this analysis, a total of 21,975 participants were included, and all estimates were weight adjusted using NHANES-specified sampling weights. Model 1 was adjusted for age (continuous), sex,, race, and FIPR. Model 2 was further adjusted for education level, BMI, marital status, smoking status, and alcohol consumption. Model 3 was additionally adjusted for histories of hypertension, high cholesterol, diabetes, CVD, and cancer. BMI, body mass index; CI, confidence interval; CVD, cardiovascular diseases; FIPR, family income to poverty ratio; HR, hazard ratio; NHANES, National Health and Nutrition Examination Survey.
